# Reduced sensitivity to minimum-jerk biological motion in autism spectrum conditions

**DOI:** 10.1016/j.neuropsychologia.2009.07.010

**Published:** 2009-12

**Authors:** Jennifer Cook, Ayse Pinar Saygin, Rachel Swain, Sarah-Jayne Blakemore

**Affiliations:** a UCL Institute of Cognitive Neuroscience, 17 Queen Square, London WC1N 3AR, UK; b City University, Optometry and Visual Science, London EC1V 0HB, UK; c University of California San Diego, Department of Cognitive Science, La Jolla, CA 93093-0515, USA

**Keywords:** Biological motion, Autism spectrum conditions, Social neuroscience, Action observation

## Abstract

We compared psychophysical thresholds for biological and non-biological motion detection in adults with autism spectrum conditions (ASCs) and controls. Participants watched animations of a biological stimulus (a moving hand) or a non-biological stimulus (a falling tennis ball). The velocity profile of the movement was varied between 100% natural motion (minimum-jerk (MJ) for the hand; gravitational (G) for the ball) and 100% constant velocity (CV). Participants were asked to judge which animation was ‘less natural’ in a two-interval forced-choice paradigm and thresholds were estimated adaptively. There was a significant interaction between group and condition. Thresholds in the MJ condition were lower than in the G condition for the NC group whereas there was no difference between the thresholds in the two conditions for the ASC group. Thus, unlike the controls, the ASC group did not show an increased sensitivity for perturbation to biological over non-biological velocity profiles.

## 1 Introduction

For many decades, researchers have used point light displays (PLDs), which consist of a few moving points and yet evoke a clear percept of a body in motion (Johansson, 1973), to study the visual perception of biological motion (see Blake & Shiffrar, 2007 for a review). The perception of biological motion is linked to the action perception network in the brain (Pelphrey & Carter, 2008; Saygin, 2007; Saygin, Wilson, Hagler, Bates, & Sereno, 2004). Autism spectrum condition (ASC) is a pervasive developmental disorder characterised by difficulties with reciprocal social interactions in addition to unusual patterns of repetitive behaviour and verbal and non-verbal communication problems (APA, 1994). Abnormalities within the action perception system have been suggested to underlie the problems with social interaction observed in ASC (Iacoboni & Dapretto, 2006; Oberman & Ramachandran, 2007; Williams, Whiten, Suddendorf, & Perrett, 2001), and it has been suggested that the perception of biological motion may be abnormal in ASC.

A number of studies have found abnormal processing of biological motion in children with ASC (Blake, Turner, Smoski, Pozdol, & Stone, 2003; Klin, Lin, Gorrindo, Ramsay, & Jones, 2009). However, since these studies have used PLDs, which require integrating the motion of multiple (typically around a dozen) points across space, it is not clear that a deficit in perceiving biological motion from PLDs is distinct from the global motion processing deficit that has also been observed in ASC (Bertone & Faubert, 2006; Milne et al., 2002; Spencer & O’Brien, 2006). Furthermore, Hubert et al. (2007) have shown that adolescents and adults with ASC showed worse performance than controls at identifying emotions from PLDs, but performance was comparable between groups when participants had to identify the portrayed action or subjective state (such as tired or bored); this finding also generalises to children (Parron et al., 2008). Hubert and colleagues suggest that biological motion processing deficits in ASC may be specific to emotional state attribution.

The current study investigated whether a biological motion processing deficit is found in ASC when the stimuli do not require global motion integration and have no emotional content. Biological motion has a characteristic velocity profile that is mathematically described by the ‘minimum-jerk model’, which is a cost function that minimises jerkiness over a specified movement trajectory (Flash & Hogan, 1985). We employed stimuli in which this minimum-jerk (MJ) velocity profile was manipulated, and a novel paradigm in which participants watched pairs of animations that showed a biological stimulus (a moving hand) or a non-biological stimulus (a falling tennis ball) moving across the screen. On each trial, the velocity profile with which each animation moved was either 100% natural motion (MJ in the biological condition; gravitational in the non-biological condition), or 100% constant velocity (CV), or some linear combination of the two extremes. In each trial, participants were shown a ‘reference’ animation, which was always a combination of 85% natural motion and 15% constant velocity, and a ‘target’ animation, in which the ratio of constant velocity to natural motion varied according to performance. The task was to judge which animation was ‘less natural’. A two-interval forced-choice adaptive staircase paradigm was employed to generate separate thresholds for the biological motion (MJ) condition and the non-biological (gravitational) condition.

## 2 Methods

### 2.1 Participants

25 participants with ASC (18 males) and 23 control participants (18 males) took part. 16 participants (14 males) from the ASC group and 16 participants (12 males) from the NC group generated adequate data required for robust perceptual threshold estimation (see below). The groups were matched for age, gender and verbal, performance and full scale IQ, as measured by the Wechsler Abbreviated Scale of Intelligence (WASI) (see Table 1).

All participants had normal or corrected-to-normal vision and were screened for exclusion criteria (dyslexia, epilepsy, and any other neurological or psychiatric conditions) prior to taking part. All participants in the ASC group had a diagnosis of autism, Asperger syndrome or ASC from a GP or psychiatrist. The Autism Diagnostic Observation Schedule (ADOS: Lord et al., 1989) was administered by a researcher trained and experienced in the use of this interview (see Table 1). We were unable to distinguish between participants with Asperger syndrome and autism, as we did not have information about early development of language and other skills in our participants. All participants gave informed consent to take part in the study, which was approved by the local ethics committee.

### 2.2 Design

Participants watched a series of visual stimuli constituting two conditions: biological (minimum-jerk; MJ) motion and non-biological (gravitational; G) motion.

### 2.3 Minimum-jerk (MJ) condition

**Fig. 1:**
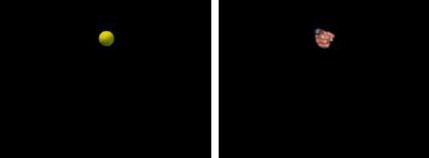
Participants watched pairs of animations that showed a biological stimulus (a hand) or a non-biological stimulus (a tennis ball) moving across the screen. On each trial, the velocity profile of the movement was either 100% natural motion (minimum-jerk in the biological condition; gravitational in the non-biological condition), or 100% constant velocity or some linear combination of the two extremes. In each trial, participants were shown a ‘reference’ animation, which was always a combination of 85% natural motion and 15% constant velocity, and a ‘target’ animation, in which the ratio of constant velocity to natural motion varied according to performance. The task was to judge which was less natural.

An image of a human hand (see Fig. 1) was programmed to make a vertical sinusoidal movement of amplitude 110 mm and frequency 0.5 Hz. The velocity profile of the stimulus was generated by motion-morphing between two movement prototypes. The velocity profile of Prototype 1 was described by a constrained MJ model (Todorov & Jordan, 1998). The model assumes that if **r**(*s*) = [*x*(*s*), *y*(*s*), *z*(*s*)] is a 3D curve describing the path of the hand during a particular trial, where *s* is the distance along the path, and tangential speed is *s*•(*t*) (*s*• is a time derivative, **r**′ is the derivative with respect to *s*, and boldface signifies vector quantities) the temporal profile of the movement will minimise the scalar function:

$$J=\int_{0}^{T}{∥\frac{d^{3}}{dt^{3}}r[s(t)]∥}^{2}dt$$

The velocity profile of Prototype 2 was described by a constant velocity (CV) vector.

### 2.4 Gravitational (G) condition

An image of a tennis ball (see Fig. 1) was programmed to make a vertical downward movement of amplitude 215 mm and frequency 1 Hz. Thus, the tennis ball appeared from the top of the screen and finished off the bottom of the screen. As in the MJ condition, the velocity profile of the stimulus was generated by motion-morphing between two prototypes of movements. The velocity profile of Prototype 1 in this condition was described by the standard equation of motion:

$$h(t)=h_{0}-0.5gt^{2}$$

where *h* is the height, *h*_0_ is the initial height, *t* is the time and *g* is the gravitational force (9.8 m/s^2^). The velocity profile of Prototype 2 was described by a CV vector.

### 2.5 Motion-morphing

In both conditions, a series of new velocity profiles was created by linear combinations of the prototype velocity profiles using the following equation:

$$\text{Motion morph}=p_{1}(\text{Prototype}\;1)+p_{2}(\text{Prototype}\;2)$$

where the weights *p*_*i*_ determine the proportion of the morph described by the individual prototype. Therefore, in each condition, stimuli were either 100% Prototype 1 (MJ or G) or 100% Prototype 2 (CV), or some linear combination of the two in which *p*_*i*_ was determined by each participant's performance on the task.

### 2.6 Procedure

In each trial participants watched a target and a reference animation, for which order was counter-balanced across trials. The reference animation was always a combination of 85% natural motion and 15% CV, but for the target animation the ratio of CV to natural motion varied according to performance. The task was to pick the less natural. Prior to testing, each participant was read instructions by the experimenter and performed at least 5 practice trials of each condition. Participants completed 6 blocks (3 of each condition) and there were 17 trials within each block. Block order was counter-balanced between participants, and participants were given breaks between blocks. The duration of the entire experiment was approximately 12 min.

### 2.7 Threshold calculation

The psychophysical threshold was determined using a two-interval forced-choice adaptive staircase procedure. The velocity profile of the reference animation was the same throughout the experiment. The velocity profile of the target animation was initially a combination of 5% natural motion (MJ or G) and 95% CV. This ratio varied according to performance. Hence, at the start of the experiment the pair of animations (reference and target) was perceptually very different in terms of their velocity profile. The proportions of each prototype in the target morph were adjusted on a trial-by-trial basis using a weighted three-down, one-up, adaptive staircase technique. The three-down, one-up transformation targets the 79.4% correct point on the psychometric function. The probability of downward movement of the adaptive track must equal the probability of an upward movement; therefore if *p* is the probability of a positive response on a given trial, then *p* × *p* × *p* must equal 0.5 hence the target probability is 3√0.5 = 0.794 (Jennings, 2005). Three correct responses in a row incurred a 0.2 (large step-size) increase in the proportion of Prototype 1 and one incorrect response led to a 0.2 decrease in the proportion of Prototype 1. Therefore, the difference between the velocity profiles of the animations converged if the participant performed well and diverged if the participant's performance declined. After the first four reversals (defined as the point at which the animations stop converging and start to diverge or vice versa), step sizes changed to 0.025 to facilitate the calculation of a fine-grained threshold. The staircase procedure was terminated after 51 trials. If the number of reversals achieved within 51 trials was greater than 12 (the potential maximum was 15) the threshold was the mean of the last 8 small-step reversals values. If the number of reversals was less than 12 but greater than 3, the threshold was the mean of all available small-step reversals.

### 2.8 Data analysis

Threshold data were analysed using a 2 × 2 mixed-model repeated-measures ANOVA with between subjects factor group (ASC vs NC) and within subjects factor condition (MJ vs G), and Bonferroni-corrected *t*-tests were used to examine simple effect differences between conditions.

## 3 Results

Data were filtered such that only thresholds based on more than three small-step reversals were included in the analysis. For the ASC group (*N* = 16), thresholds in the MJ condition were estimated from (mean) 5.19 (±2.61 SD) small-step reversals and from 4.69 (±1.92) small-step reversals in the G condition. For the NC group (*N* = 16), thresholds were estimated from 5.88 (±1.59) reversals in the MJ condition and 5.56 (±1.86) in the G condition. There was no significant difference between groups in the number of reversals used for threshold calculation in either condition (MJ condition: *t*(30) = −0.9, *p* = 0.38; G condition: *t*(30) = −1.31, *p* = 0.2). In addition, the number of reversals did not differ between conditions for each group (ASC: *t*(15) = 0.6, *p* = 0.56; NC: *t*(15) = 0.42, *p* = 0.68).

**Fig. 2:**
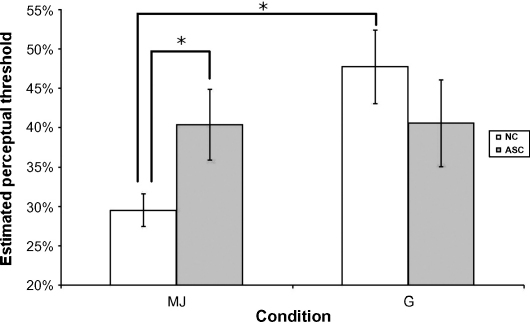
There was a significant interaction between group and condition driven by lower thresholds in the MJ condition than in the G condition for the NC group but not for the ASC group.

ANOVA revealed a significant main effect of condition (*F*(1,30) = 4.558, *p* < 0.05) and a significant interaction between condition and group (*F*(1,30) = 4.37, *p* < 0.05). There was no significant main effect of group (*F*(1,30) = 0.17, *p* = 0.68). The interaction was driven by a significant difference between the groups in the MJ condition (mean MJ thresholds for NC: 0.29 ± 0.02; ASC: 0.40 ± 0.05; *t*(30) = 2.197, *p* < 0.05) but not in the G condition (mean G thresholds for NC: 0.48 ± 0.05; ASC: 0.41 ± 0.05; *t*(30) = −1, *p* = 0.32). Thresholds in the MJ condition were significantly lower than in the G condition for the NC group (*t*(15) = −3.127, *p* < 0.01), whereas there was no significant difference between conditions for the ASC group (*t*(15) = −0.03, *p* = 0.97) (see Fig. 2).

## 4 Discussion

To our knowledge, this is the first study to have measured thresholds for detection of perturbations to biological and non-biological motion. The thresholds reflect the amount of CV motion necessary to perturb a natural motion animation such that, if presented with the perturbed animation and a natural motion exemplar, the participant can no longer discriminate the less natural. Low thresholds, therefore, reflect high sensitivity to CV perturbations whereas high thresholds reflect low sensitivity to CV perturbations. The NC group exhibited mean thresholds for MJ motion of 30%, indicating that, on average, 70% of the velocity profile must be MJ for the target to be discriminated as ‘less natural’ than the reference (which contained 85% MJ). In contrast, the mean non-biological (G) motion threshold was 48% for the NCs. Thus, on average for the NCs, 52% of the velocity profile must be G for the target to be discriminated as ‘less natural’ than the reference (which contained 85% G). This suggests that the NC group was more sensitive to CV perturbations to the velocity profile of biological (MJ) motion than to perturbations to non-biological (G) motion. In the ASC group, mean thresholds were similar for both biological motion and non-biological motion (approximately 40% in both conditions). This indicates that, whilst the NC group was particularly sensitive to changes in the velocity profile of biological relative to non-biological motion, this increased relative sensitivity to biological motion was not found in the ASC group.

The difference between the two groups’ perceptual thresholds appears to be specific to biological motion since there was no difference between thresholds for non-biological motion. Since both groups obtained similar thresholds in the G condition, it is unlikely that the difference between groups in the MJ condition was due to differences in the interpretation of the task instructions, or attention. The atypical biological motion processing found here is in line with previous findings of abnormal biological motion processing in children with ASC (Blake et al., 2003; Klin et al., 2009). However, the task used in the current study did not require global motion integration or processing of emotional content (Bertone & Faubert, 2006; Hubert et al., 2007; Milne et al., 2002; Spencer & O’Brien, 2006). Therefore, our data provide evidence for a biological motion processing deficit in ASC that cannot be explained by the need to integrate motion signals across space or the need to process the emotional content of the stimuli.

Recently, Klin and colleagues found that 2-year olds with ASC spend less time attending to biological motion than do typically developing controls (Klin et al., 2009). It may be the case that the reduced sensitivity to biological motion in an adult sample with ASC, as found in the current study, is a developmental consequence of reduced observation of biological motion and hence abnormal learning about the kinematics of human movement. It would be interesting to investigate whether young children with ASC show abnormalities in the perception of MJ motion, using stimuli that do not require global motion integration similar to those used in the current study.

## Figures and Tables

**Table 1:** Participant details. Mean (±SD) scores for age, IQ and ADOS are provided. Note that IQ scores were available for only 10 out of 16 NC participants.

	ASC	NC	Group comparison
*N*	16	16	
Gender (M:F)	14:2	12:4	
Age in years	34.1 (12.4)	33.3 (12.2)	*t*(30) = 1.72; *p* = 0.86
Verbal IQ	117 (16.5)	118 (11.64; *N* = 10)	*t*(24) = 0.43; *p* = 0.87
Performance IQ	109 (12.9)	113 (11.55; *N* = 10)	*t*(24) = 0.60; *p* = 0.44
Full scale IQ	114.8 (15.56)	113 (15.06; *N* = 10)	*t*(30) = 0.34; *p* = 0.74
ADOS total score	7.06 (3.47)	NA	
ADOS RSI	5.06 (2.35)	NA	
